# Assessing Potential Exemplars in Reducing Zero-Dose Children: A Novel Approach for Identifying Positive Outliers in Decreasing National Levels and Geographic Inequalities in Unvaccinated Children

**DOI:** 10.3390/vaccines11030647

**Published:** 2023-03-14

**Authors:** Nancy Fullman, Gustavo C. Correa, Gloria Ikilezi, David E. Phillips, Heidi W. Reynolds

**Affiliations:** 1 Exemplars in Global Health, Gates Ventures, 2401 Elliott Ave, Seattle, WA 98121, USA; 2 Gavi, the Vaccine Alliance, Chemin du Pommier 40, Le Grand-Saconnex, 1218 Geneva, Switzerland

**Keywords:** immunization, vaccines, zero-dose children, equity

## Abstract

Background: Understanding past successes in reaching unvaccinated or “zero-dose” children can help inform strategies for improving childhood immunization in other settings. Drawing from positive outlier methods, we developed a novel approach for identifying potential exemplars in reducing zero-dose children. Methods: Focusing on 2000–2019, we assessed changes in the percentage of under-one children with no doses of the diphtheria–tetanus–pertussis vaccine (no-DTP) across two geographic dimensions in 56 low- or lower-middle-income countries: (1) national levels; (2) subnational gaps, as defined as the difference between the 5th and 95th percentiles of no-DTP prevalence across second administrative units. Countries with the largest reductions for both metrics were considered positive outliers or potential ‘exemplars’, demonstrating exception progress in reducing national no-DTP prevalence and subnational inequalities. Last, so-called “neighborhood analyses” were conducted for the Gavi Learning Hub countries (Nigeria, Mali, Uganda, and Bangladesh), comparing them with countries that had similar no-DTP measures in 2000 but different trajectories through 2019. Results: From 2000 to 2019, the Democratic Republic of the Congo, Ethiopia, and India had the largest absolute decreases for the two no-DTP dimensions—national prevalence and subnational gaps—while Bangladesh and Burundi registered the largest relative reductions for each no-DTP metric. Neighborhood analyses highlighted possible opportunities for cross-country learning among Gavi Learning Hub countries and potential exemplars in reducing zero-dose children. Conclusions: Identifying where exceptional progress has occurred is the first step toward better understanding how such gains could be achieved elsewhere. Further examination of how countries have successfully reduced levels of zero-dose children—especially across variable contexts and different drivers of inequality—could support faster, sustainable advances toward greater vaccination equity worldwide.

## 1. Introduction

The expansion of routine immunization is heralded as a global success story [1], enabling greater survival and improved child health worldwide [2]. Nevertheless, an estimated 25 million children were un- or under-vaccinated in 2021 [3], with many facing compounding barriers in vaccine access, availability, and demand. The ongoing COVID-19 pandemic has contributed to at least some of today’s gaps in childhood vaccination [3], with estimates of under-one children without any doses of the diphtheria–tetanus–pertussis vaccine (no-DTP) rising from 10% prevalence in 2019 to 14% in 2021 [3]. Communities with high levels of unvaccinated or “zero-dose children” often face myriad vulnerabilities [4,5,6,7], such as residing in highly remote areas or informal settlements in cities [7,8,9]; being affected by displacement and/or prolonged conflict or unrest [7,8]; longstanding poverty and/or societal neglect [4]; or some constellation of these factors. Subsequently, optimally identifying where and how to better reach zero-dose children will likely require a combination of context-specific strategies and broader investments to address persisting structural challenges.

Over the last few years, a growing body of research has sought to assess characteristics of zero-dose children and their families or households, as well as potential drivers of high zero-dose prevalence at different geographic levels [4,5,6,7,8,10,11,12,13,14,15,16,17]. Past work has found that zero-dose children experience a higher odds of missing or lacking access to other types of primary care services [6,11,12], while their mothers were more likely to have no antenatal care visits and not deliver at a health facility [11,12]. Lower levels of household wealth, educational attainment, and measures of women’s empowerment also have been associated with higher levels of zero-dose children [4,10,16,17]. Gender-based inequalities, which span from differential rates of immunization by infant gender and gender-related barriers related to who can seek or provide vaccination services [16,18], emphasize the complex yet crucial role that gender plays in a country and/or community [19]. Prior studies have found ethnic disparities [15], as well as differences by religious affiliation [13,20], among children who have received no doses of DTP, though the exact nature of these relationships varied by country. Quantifying these risk factors and determinants of zero-dose children can provide critical program inputs, spanning from identifying key barriers to service access [7,18] to honing in on what sociocultural forces may be negatively affecting vaccine sentiments and trust [18,19,20]. However, exclusively focusing on zero-dose risk profiles and factors associated with higher rates of unvaccinated children may miss important lessons around successful approaches to addressing inequalities in childhood immunization. Accordingly, also understanding what has worked to improve childhood vaccination can inform program and policy adaptations tailored for reaching zero-dose children.

Positive outlier, or so-called ‘positive deviance’, methodologies have been used at the unit or organizational level in healthcare settings [21,22,23], as well as for more population-level contexts [24,25,26,27,28], to generate or strengthen the evidence base around what works to improve key health priorities. While the exact approaches toward this type of research and synthesis vary, they usually espouse a shared premise: knowledge and implementation strategies around achieving success or progress exist from places or contexts where such success or progress have been previously attained [21]. As a result, identifying and then examining what contributes to exceptional performance or progress can offer actionable insights into what policies and practice could be adapted for similar impact elsewhere. For instance, the *Good Health at Low Cost* case studies first in 1985 [26] and then in 2013 [27], sought to synthesize how and why countries or regions achieved substantial advances in several health indicators compared to their peers with similar income and demographic profiles; in 2018, the World Bank took a similar approach for understanding rapid progress on universal health coverage measures and facilitating shared learning opportunities across countries [28]. In 2016, the Global Burden of Disease study developed analyses to compare country-level performance on various health metrics relative to changes in sociodemographic development [29,30,31]; such findings emphasized that important health program and policy lessons could be learned from countries where achievements exceeded expected levels or trends on the basis of sociodemographic improvements alone. Lastly, the Exemplars in Global Health (EGH) program has sought to synthesize key lessons and strategies used by countries that attained exceptional progress in health—exemplars—through mixed-methods research and engagement with partners [32,33,34,35]. As highlighted by past and current work on positive outliers, such analyses can foster opportunities for cross-country learning and exchange around successful policy or programmatic approaches for a given health challenge. With more learning agendas and priority-setting around zero-dose children for both national and global initiatives (e.g., Immunization Agenda 2030 [IA2030] [36] and Gavi 5.0 [37]), adopting a positive outlier lens toward country progress in reducing zero-dose children could further inform key immunization program and policy efforts.

With this study, we develop a novel approach for identifying positive outliers in reducing zero-dose children over time. This analysis currently takes a geographic focus, one of many important dimensions of inequality, by comparing patterns in both national and subnational declines in the percentage of under-one children with no doses of DTP (no-DTP) from 2000 to 2019 among 56 low- and lower-middle-income countries (LMICs). Based on this approach, identified ‘exemplar’ countries or subnational locations that substantially reduced zero-dose children could be targeted for further examination into the policy or program factors behind such gains.

## 2. Materials and Methods

### 2.1. Data

We used estimates of DTP1 among children under 1 year of age at the national and second administrative levels from the Institute for Health Metrics and Evaluation (IHME). The methods used to estimate DTP1 at different geospatial resolutions are detailed elsewhere [38]; in brief, DTP1 coverage estimates were derived from georeferenced household surveys and modeled using Bayesian geostatistical methods for 106 countries at the first and second administrative levels from 2000 to 2019. We opted to use these spatially modelled estimates over alternative sources (e.g., administrative data) to maximize both the potential number of countries included and comparability of estimates across locations. We subtracted DTP1 estimates from 100% to reflect the percentage of under-one children with no doses of DTP, or no-DTP prevalence—a commonly used indicator for zero-dose children [10,36].

For this analysis, we focused on 56 LMICs (Table 1). These countries were selected on the following criteria: (1) designation of low- or lower-middle income for fiscal year 2020 by the World Bank [39] or having received support from Gavi, the Vaccine Alliance as of 2018 [40]; (2) availability of both national and subnational no-DTP estimates at the second administrative level from 2000 to 2019; (3) not being classified as a post-transition middle-income country by Gavi and inclusion as part of Gavi’s zero-dose segmentation country groups [41]. Supplementary Table S1 includes the full list of initially considered countries and those excluded from the current analysis.

### 2.2. Analysis

We conducted three analyses to characterize potential exemplars in reducing zero-dose children over time, as summarized below. R version 4.2.1 was used for data processing, analyses, and visualizations [42].

**Quantifying changes in zero-dose children across geographies.** We assessed changes in the percentage of under-one children without any doses of DTP (no-DTP) between 2000 and 2019 across two geographic dimensions: (1) national levels; (2) subnational gaps among second-level administrative units. For the latter—subnational gaps—we used the 5th and 95th percentile values of the prevalence of no-DTP children estimated across second-level administrative units and computed the difference for a given country–year. We opted to use the 5th and 95th percentiles rather than absolute minimum and maximum values of no-DTP prevalence to offset the potential for undue influence of outliers for a given subnational unit–year. Furthermore, how countries define second-level administrative units widely varies (e.g., 10 or fewer units in Comoros, São Tomé and Príncipe, and Lesotho to 774 local government areas (LGAs) in Nigeria); using percentiles to define subnational gaps may also help mitigate the degree to which having more (or fewer) administrative units could affect measures of subnational inequality.

**Identifying potential exemplars in reducing zero-dose children.** Second, countries with the largest declines for *both* no-DTP metrics between 2000 and 2019 were considered as potential exemplars in reducing zero-dose children. Prior research conducted under the EGH program has typically used one progress measure per geographic unit [32,34,35], and then benchmarked changes against indicators of sociodemographic development. Because many locations with the highest levels of unvaccinated children face compounding vulnerabilities [4], any investments in reaching zero-dose children should also correspond with action to address disparities in immunization rates. Our approach to operationalizing this pro-equity lens from a geographic perspective was equally weighting reductions at the national level and subnational differences for no-DTP. In other words, a country that achieved marked national reductions in no-DTP prevalence without corresponding declines in subnational gaps should not be considered a potential exemplar in reducing zero-dose children. 

We ranked each country ordinally, 1 to 56, based on their national and subnational reductions in no-DTP prevalence from 2000 to 2019, with 1 being the largest reduction and 56 being the smallest reduction or, if applicable, the largest increase since 2000. We took the mean of those rankings to identify which countries had achieved the most progress across both geographic dimensions. We applied these rankings and calculations for absolute and relative progress separately: computing percentage point changes for absolute progress from 2000 to 2019 and then percentage change from 2000 to 2019 for relative progress. We opted to consider both progress metrics—absolute and relative progress—as they could better represent a range of successful approaches used to reduce no-DTP prevalence from different starting points (i.e., higher and lower absolute levels of no-DTP children in 2000), and thus likely mirror different stages of immunization delivery needs and strategies.

**Comparing divergent no-DTP trajectories since 2000 for select locations**. Third, we conducted so-called “neighborhood analyses” for select countries, comparing them to other countries that had similar levels for both no-DTP measures in 2000 but different trajectories through 2019. Such analyses are thought to be supportive of potential cross-location learning and knowledge translation around what could work to address zero-dose challenges when starting from similar baseline levels of no-DTP prevalence. At the country-level, we focused on Nigeria, Mali, Uganda, and Bangladesh—the four countries selected for the Gavi Learning Hubs [43] and sought to match a “neighbor” exemplar to each country. Further detail on the Gavi’s Learning Hub initiative is available elsewhere [43]; in brief, these four countries were selected on the basis of zero-dose metrics (i.e., high absolute numbers or prevalence of zero-dose children) as well as variations in zero-dose prevalence across geographic locations and among key populations that experience higher rates of no vaccination (i.e., rural, urban poor, refugeed, or conflict settings). A primary objective of the Learning Hubs is to support deeper assessment and engagement to improve monitoring and measurement systems, and to enable learning about what works programmatically to reach unvaccinated children and missed communities.

## 3. Results

### 3.1. Quantifying Changes in No-DTP Children from 2000 to 2019

**Figure 1:**
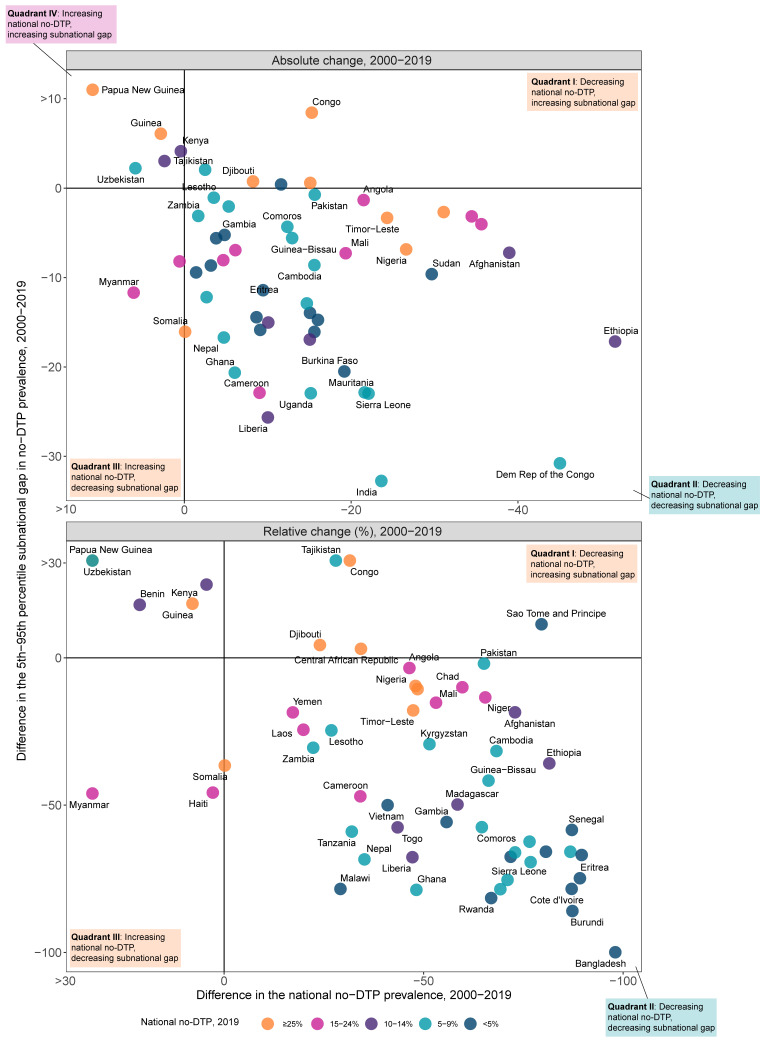
Comparing changes in no-DTP prevalence, nationally and for subnational gaps, from 2000 to 2019 for 56 LMICs. Countries are color-coded by national estimates of no-DTP in 2019.

Among the 56 LMICs included in this analysis, 44 (78.6%) had some kind of reduction in both national levels of no-DTP and subnational gaps in no-DTP prevalence between 2000 and 2019 (Figure 1; Table 2). In contrast, five countries—Benin, Kenya, Guinea, Papua New Guinea, and Uzbekistan—had at least some increase in both estimated national and subnational gaps in no-DTP prevalence. Five countries decreased national no-DTP levels between 2000 and 2019, but in tandem saw subnational gaps increase to some degree: Congo (an 8.4 percentage-point rise); Tajikistan (2.1 percentage points); Djibouti (0.7 percentage points); Central African Republic (0.6 percentage points); and São Tomé and Príncipe (0.4 percentage points). Two countries—Haiti and Myanmar– had the national percentage of under-one children with no DTP doses at least somewhat increase since 2000 while subnational gaps declined; this was particularly pronounced for Myanmar (a 6.1 percentage-point rise).

### 3.2. Identifying Potential Exemplars in Reducing Zero-Dose Children since 2000

**Figure 2:**
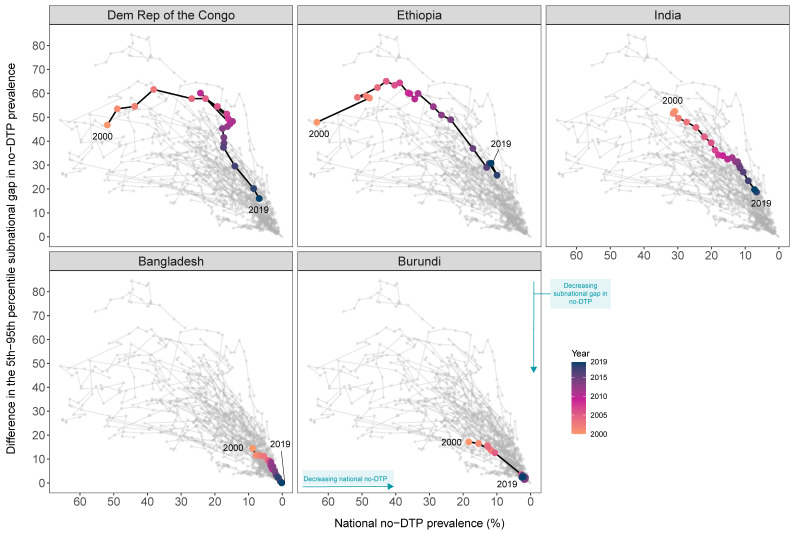
Comparing no-DTP trajectories for potential exemplars in reducing zero-dose children, 2000 to 2019. National no-DTP prevalence is represented on the x-axis and the subnational gap (as measured by the difference between the 5th and 95th percentile no-DTP prevalence across second-level administrative units) is represented on the y-axis. Each corner represents an extreme for each of these no-DTP metrics, with the lower right-hand corner—low national no-DTP prevalence and low subnational inequality—being the direction in which every location should strive to reach to equitably reduce no-DTP prevalence. Trends in the two no-DTP metrics for the potential exemplars are highlighted in black, with each circle representing a year from 2000 to 2019 that is color-coded from orange (2000) to blue (2019). The light gray trajectories represent the other 51 countries in this analysis.

***Absolute progress.*** The DRC, Ethiopia, and India registered the largest absolute reductions in national no-DTP prevalence and subnational gaps from 2000 to 2019 (Figure 2; Table 2). Supplementary Figure S1A–C show both national and subnational no-DTP trends over time for each country.

In 2000, 51.9% of under-one children had no doses of DTP in the DRC nationally, with the country experiencing a 46.8 percentage-point gap between the territories with 5th and 95th percentiles for no-DTP prevalence (i.e., 28.5% to 75.3%). By 2019, national no-DTP prevalence levels fell to 6.9%, a 45.0 percentage-point decline. The DRC’s subnational gaps narrowed by 30.8 percentage-points by 2019, decreasing to a total of 16.0 percentage points across the 5th and 95th percentiles of territories (i.e., 1.5% to 17.5%). As highlighted by Figure 2 and Figure S1A, subnational gaps started narrowing faster from 2015–2019 than in previous time periods.

For Ethiopia nationally, 63.4% of under-one children lacked any doses of DTP in 2000, but no-DTP prevalence fell 51.6 percentage points to 11.7% by 2019. Across its zones, Ethiopia had a 47.9 percentage-point gap between the 5th and 95th percentile levels of no-DTP prevalence in 2000, spanning from 39.6% to 87.5%. This subnational gap decreased by 17.2 percentage points by 2019, to a 30.8 percentage-point difference between the 5th and 95th percentile no-DTP levels across zones (i.e., 1.6% to 32.4%). However, amid such marked gains over the last 19 years, Ethiopia’s reductions in subnational gaps have stagnated from 2016–2019 (Figure 2 and Figure S1B).

In India, national no-DTP prevalence was 30.9% in 2000 with a 52.5 percentage-point gap between the 5th and 95th percentile for no-DTP levels across districts (i.e., 7.2% to 59.7%). By 2019, 7.2% of under-one children had no doses of DTP in India nationally, a 23.6 percentage-point decline. Subnational gaps in India decreased 32.7 percentage points between 2000 and 2019, falling to 19.7 percentage-point difference in 2019 (i.e., 1.6% to 21.3%). Although overall subnational gaps have narrowed (Figure 2 and Figure S1C), several districts still exceeded 30% of under-one children with no doses of DTP in 2019.

***Relative progress***. As measured by the percentage change in no-DTP metrics between 2000 and 2019, Bangladesh and Burundi achieved the largest relative reductions in no-DTP prevalence (Figure 2; Table 2). National and subnational no-DTP trends are illustrated in Supplementary Figure S1D,E. 

In 2000, estimated national prevalence of no-DTP was 8.8% in Bangladesh, already below the 10% target set forth by the Global Vaccine Action Plan for 2020 [44]. However, by 2019, the percentage of under-one children with no doses of DTP fell to 0.8% in Bangladesh, a 98.0% decline since 2000. Subnational gaps in Bangladesh fell by 99.9% since 2000, narrowing from a 14.4 percentage-point difference for the 5th and 95th percentiles across districts in 2000 (i.e., 3.3% to 17.7%) to approximately 0.01 percentage-points in 2019. Absolute subnational gaps began narrowing faster after about 2012 (Supplementary Figure S1D).

For Burundi, national no-DTP estimates were 18.4% in 2000, but decreased to 2.3% in 2019—an 87.3% reduction. Across Burundi’s communes in 2000, there was a 17.1 percentage-point difference for the 5th and 95th percentiles in no-DTP prevalence (i.e., 10.2% to 27.3%). By 2019, this subnational gap fell to 2.4 percentage points, representing an 85.9% decline across communes at the 5th and 95th percentile (i.e., 1.5% to 3.9%). Progress accelerated after 2005 (Figure 2, Supplementary Figure S1E), when the country’s 13-year civil war ended [45].

Table 2 details these estimates for 2000, 2019, and across change metrics for all 56 countries, while Figure 2 depicts trajectories for no-DTP across national levels and subnational gaps for potential exemplars in reducing zero-dose children for each year between 2000 to 2019.

### 3.3. Comparing Divergent No-DTP Trajectories since 2000 for Select Locations

**Figure 3:**
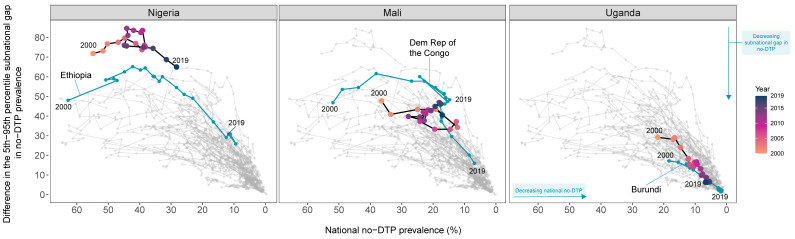
Comparing Gavi Learning Hub country no-DTP trajectories since 2000 to potential exemplars in reducing zero-dose children. Bangladesh, a Gavi Learning Hub country, was identified as potential exemplar based on its marked progress on relative no-DTP metrics of change (Figure 2). Accordingly, we focus on Nigeria, Mali, and Uganda here. National no-DTP prevalence is represented on the x-axis and the subnational gap (as measured by the difference between the 5th and 95th percentile no-DTP prevalence across second-level administrative units) is represented on the y-axis. Each corner represents an extreme for each of these no-DTP metrics, with the lower right-hand corner—low national no-DTP prevalence and low subnational inequality—being the direction in which every location should strive to reach to equitably reduce no-DTP prevalence. Trends in the two no-DTP metrics for the Gavi Learning Hub countries are highlighted in black, with each circle representing a year from 2000 to 2019 that is color-coded from orange (2000) to blue (2019). The teal trends represent trajectories for the potential exemplars in reducing zero-dose children based on their absolute or relative progress since 2000. The light gray trajectories represent the other countries in this analysis.

Focusing on the four Gavi Learning Hub countries—Nigeria, Mali, Uganda, and Bangladesh—we mapped their no-DTP trajectories from 2000 to 2019 against potential exemplars in reducing zero-dose children (Figure 3); the exception was Bangladesh, which achieved among the largest relative reductions in national no-DTP prevalence and subnational gaps since 2000. Accordingly, Figure 3 excludes Bangladesh.

For Nigeria, Ethiopia was its closest ‘neighbor’ in terms of national no-DTP prevalence in 2000—55.5% in Nigeria and 63.4% in Ethiopia—with diverging no-DTP trajectories through 2019 (i.e., 28.9% in Nigeria and 11.7% in Ethiopia). From 2000 to 2019, Nigeria consistently had among the highest subnational no-DTP disparities in the world; even in 2000, when Ethiopia had the fourth highest subnational gap in no-DTP among included countries (47.9 percentage points; Table 2), Nigeria’s subnational gap was more than 20 percentage points higher (71.7; Table 2). Nonetheless, given how Ethiopia markedly reduced no-DTP subnational gaps at the same time trends in Nigeria’s subnational disparities more or less stagnated, they may be well-aligned for cross-country learning.

For Mali, the DRC was its closest ‘neighbor’ for subnational no-DTP prevalence gaps in 2000, with Mali experiencing a 47.8 percentage-point gap and the DRC having a 46.8 percentage-point disparity. By 2019, Mali still had a subnational gap exceeding 40 percentage points (40.5, Table 2) while the DRC reduced its subnational gap to 16.0 (Table 2). National no-DTP prevalence was more variable for Mali and the DRC, with Mali’s national no-DTP levels registering far lower than the DRC’s in 2000 (36.4% and 51.9%, respectively) but then only moderately declining to 17.1% by 2019. In contrast, the DRC’s national no-DTP prevalence decreased to 6.9% in 2019. Yet the DRC’s no-DTP metrics from 2010–2015—the time before the country accelerated no-DTP reductions—parallel Mali’s 2019 no-DTP measures. Accordingly, this more recent time period may support optimal cross-country learning for Mali. 

For Uganda, Burundi aligned most closely to its 2000 no-DTP measures but showed divergences by 2019. In 2000, national no-DTP prevalence was 21.9% in Uganda and 18.4% in Burundi; by 2019, their no-DTP estimates were 6.7% and 2.3%, respectively (Table 2). Subnational gap trends were less similar for these two countries, with Burundi’s no-DTP subnational gap in 2000 being narrower (17.1 percentage points) than that of Uganda’s (29.2 percentage points). Each country recorded sizeable declines in subnational no-DTP gaps, with Uganda’s falling to 6.3 percentage points and Burundi’s to 2.4. In many ways, both Uganda and Burundi could offer meaningful lessons around reducing subnational disparities among unvaccinated children.

## 4. Discussion

With this analysis, we offer a novel application of positive-outlier methods for identifying potential exemplars in reducing zero-dose children since 2000. The DRC, Ethiopia, and India showed among the largest absolute declines in both national no-DTP prevalence and subnational gaps between 2000 and 2019, while Bangladesh and Burundi demonstrated the largest percentage decreases in national no-DTP prevalence and subnational gaps during that time. Given the range of starting points, local contexts, and health system structures in these five countries, it is quite possible that the strategies used, and corresponding lessons learned in improving childhood vaccination—specifically around expanding service reach to unvaccinated children—may be applicable (or at least adaptable) to other settings. As highlighted by the so-called “neighborhood” analysis, comparing divergent no-DTP trajectories among peer locations could support deeper study around what catalyzed faster progress for some places—and how those lessons could be applied elsewhere. The combination of this positive outlier methodology and cross-country platforms supported by the Gavi Learning Hubs offers unique opportunities to better understand ‘what works’ for accelerating progress in reaching unvaccinated children worldwide.

Considering positive outliers—or potential exemplars—in reducing no-DTP prevalence for both absolute and relative progress can better reflect the range of successful strategies implemented from a range of different no-DTP prevalence starting points. After all, the types of programmatic and policy decisions that may occur when more than 30–50% of under-one children have had no doses of DTP could differ from those occurring when high zero-dose communities are more clustered and national levels of no-DTP are well below 10%. In health service delivery, these differences may unfold around more widespread intervention introduction and scale-up activities (e.g., addressing key infrastructure and personnel gaps that would otherwise impede adoption; mass mobilization and campaign-style outreach efforts) versus more tailored service provision to individuals or communities who still lack access to or demand for an intervention (e.g., hard-to-reach and hard-to-vaccinate populations [46]). For instance, in 2000, the DRC and Ethiopia started among the highest national levels of no-DTP observed across included countries in this study, as well as moderate-to-high levels of subnational gaps. Better understanding how the DRC and Ethiopia substantially reduced no-DTP metrics by 2019 could strengthen strategies adapted for countries that started at similar no-DTP measures in 2000 but had minimal or less pronounced reductions (e.g., Nigeria, Chad, Somalia). 

Despite their marked progress since 2000, further improvements in vaccination reach and uptake are needed in the DRC, Ethiopia, India, and other countries still experiencing large populations of unvaccinated children. Accordingly, it is possible that lessons learned from countries with exceptional relative reductions from 2000 to 2019 could be applicable to countries such as the DRC, Ethiopia, and India today; after all, 2019 no-DTP estimates for the latter countries are quite similar to the 2000 estimates for countries such as Bangladesh and Burundi. For Bangladesh, reductions in national no-DTP and subnational gaps nearly paralleled each other time, charting a path toward nearly 0% no-DTP nationally and negligible subnational differences by 2019. These trends may reflect the country’s concerted efforts to better reach rural communities with lower levels of vaccination [47], among other immunization and primary care strengthening interventions. For Burundi, levels of and subnational gaps in no-DTP markedly declined after the end of its civil war in 2005 [45]. From 2006 to 2010, Burundi adopted nationwide performance-based financing initiatives focused on improving child and maternal care [48], actions that have been associated with higher vaccination rates, particularly among the poor [49]. To better understand how different interventions and strategies may optimally align with current needs and barriers to vaccination, it is crucial to more deeply examine the programs and contexts in which past gains have occurred.

There is ample opportunity—and need—to characterize what drives successful vaccine delivery and uptake across the spectrum of past and current challenges, particularly around vaccination inequalities. One key consideration that emerged from this analysis involves the pathways by which no-DTP changed both nationally and sub-nationally from 2000 to 2019. Particularly among countries that started with higher levels of no-DTP (e.g., Nigeria and Mali; Ethiopia, and the DRC), subnational gaps often remained unchanged or increased while national no-DTP prevalence began improving. Such pathways suggest that explicit equity program targets and implementation practices may not occur until later. Other countries, including India and Bangladesh, had more consistent declines for both metrics from 2000 to 2019—a potential signal into the ways in which countries are concurrently addressing both national vaccination priorities and at least geographic inequalities. Nonetheless, it is also possible that countries such as India and Bangladesh experienced similar pathways of minimal changes in or rising subnational inequality amid decreasing national no-DTP prior to 2000. Developing a more formalized characterization or framework around ‘pathways of progress’ toward greater vaccination equity should be considered in future studies, both by geography and across other crucial factors (e.g., gender, wealth, education, religion, ethnicity). 

While assessing progress metrics is a necessary first step to better identify potential exemplars in reducing zero-dose burdens, they alone cannot shed light on what countries have executed and how such actions were associated with further improvements. Formally applying methods such as that of the EGH program, with qualitative examination of policy and programs alongside quantitative analyses around drivers of progress [32], should be prioritized for countries and/or subnational locations with notable advances in reducing zero-dose children. Furthermore, the learning and evaluation platform offered through the Gavi Learning Hubs [43], wherein characteristics of immunization programs and factors contributing to their impact will be examined in prospective manner with country researchers and leadership, will enable greater cross-country or subnational engagement around what works to reach unvaccinated children across contexts. This is particularly important for larger countries where subnational locations started at similar starting points but experienced different trajectories over time. For instance, in Nigeria, bordering states Kaduna and Plateau had fairly high levels of no-DTP prevalence in 2000 (69.4% and 50.6%, respectively; Supplementary Figure S2A). By 2019, Plateau decreased its no-DTP prevalence to 14.5%, whereas Kaduna reduced no-DTP prevalence to 31.9%. Another pair of bordering states—Kogi and Enugu—had no-DTP prevalence of 48.1% and 40.7% in 2000; by 2019, Engu recorded a much larger decline by 2019 (to 7.5%) whereas Kogi still exceeded 20% no-DTP prevalence. In Ethiopia, three regions—Afar, Somali, Benshangual-Gomez—had the country’s highest no-DTP prevalence in 2000, at 75% or higher, followed by Oromia (69.3%) (Supplementary Figure S2B). By 2019, Benshangual-Gomez and Oromia reduced regional levels of no-DTP to 8.3% and 11.1%. Although Afar and Somali also recorded substantive declines in overall no-DTP prevalence, each region still had no-DTP prevalence exceeding 25%—and experienced widening gaps in no-DTP among zones. Given these trends and patterns, it is likely that many countries—especially larger ones—could benefit from so-called neighborhood analyses and positive outlier research at the subnational level.

Past work has sought to synthesize and/or assess particular characteristics of successful immunization programs; nonetheless, few studies have expressly focused on both zero-dose children and incorporating mixed-methodologies with a positive outlier lens. For example, qualitative research in Senegal, Zambia, and Nepal points to factors including strong community engagement, integrated delivery, adaptive service provision, and robust data systems as central to improving and/or maintaining high levels of DTP1 and/or DTP3 [35,50,51,52]. However, the degree to which these approaches are fully transferable to communities with high zero-dose burdens remains unclear. Integrated service delivery, particularly for key primary care interventions for mothers and infants, may have an important role in addressing zero-dose burdens given the high overlap of missing vaccine doses with other essential health services [11]. Strengthening community engagement may require taking a longer-term lens and multifaceted investments, especially in areas of prolonged conflict and/or distrust of health systems and providers. Innovative programs such as the DRC’s Mashako Plan, which was launched in 2018 and has sought to improve vaccination completion rates among select provinces through a mixture of supervision support, supply chain improvements, and monitoring efforts [53], may also provide valuable implementation lessons for countries with equally large and/or dispersed populations.

It is worth noting that declines in no-DTP prevalence—and thus increased coverage of DTP1, a marker of program reach—do not inherently equate to gains in broader program retention or complete immunization. For instance, in much of Ethiopia, DTP3 coverage has not improved in parallel amid sizeable increases in DTP1 [7,54,55]. This means that while more children are being reached by vaccination services—an unequivocally crucial milestone—an increasing percentage of them remain under-vaccinated and thus may still be vulnerable to preventable disease. Although some parts of immunization programs can support both vaccination initiation and completion well (e.g., sufficient availability of qualified health workers, strong supply, and cold chain systems), other factors can differentially affect how or whether children finish vaccination series after receiving their first doses [7,56,57]: the availability of defaulter tracking systems, provider-client relationships and trust, flexibility in scheduling for multiple vaccine doses and/or other health services, among others. As global immunization agendas such as IA2030 [36] and Gavi 5.0 [37] rightly bring more attention to zero-dose populations and programs strategies to reach them, it is crucial that political and funding commitments around addressing gaps in under-vaccination also are maintained.

### Limitations

This analysis is subject to a number of limitations. First, this study focuses on changes in geographic inequalities at the second administrative level or higher, which results in representing only one of many critical factors that contribute to inequities in immunization delivery [19]. While geographic location can serve as a proxy for determinants also associated with location (e.g., district-level program funding levels, relative remoteness) [58], geography on its own cannot appropriately approximate the mechanisms by which gender, ethnicity, education, wealth, religious affiliation, and other individual, household, or community characteristics affect childhood vaccination [4,8,12,13,14,16,18]. It is also very possible that reductions in geographic inequalities do not consistently correspond with decreases in vaccination inequalities by these other key drivers of disparities across locations or do so consistently over time. Accordingly, it is critical to prioritize future research and analyses that explicitly assess how these trends in inequality may correlate with each other.

Second, focusing on the second-administrative level likely masks important differences experienced at more granular levels (e.g., within communities), [38,54] and thus potentially could obscure a more nuanced understanding of the localized sociocultural and/or economic contributors to higher levels of zero-dose children. Future analyses should explore alternative geographic levels or areal operationalizations (e.g., 5 × 5 km pixel estimates rather than administrative boundaries) to further characterize the distribution and magnitude of vaccination inequalities in a given location.

Third, country-to-country comparisons of subnational gaps and changes in these gaps over time may be affected by a country’s total number of second-level administrative units rather than meaningful differences in vaccination equity at comparable areal units. For instance, subnational gaps in no-DTP may seem higher among in a country divided into more second-level administrative units than those with fewer units [59]. At least for the present analysis, having more (or fewer) second-level administrative units does not appear to be strongly related to 5th/95th percentile gap measures (i.e., *r* = 0.47 in 2000 and *r* = 0.38 in 2019) or change metrics from 2000 to 2019 (i.e., *r* = −0.17 for absolute change and *r* = 0.03 for percentage change). Since first- or second-level administrative units are often meaningful for health program implementation (e.g., district health authorities), we viewed using country administrative units as having more benefits and relevance than the potential drawbacks around variable subnational geographies. However, exploring alternative units of analysis (e.g., standardized pixel units) could be beneficial for future work.

Fourth, we opted to use estimates from IHME for this analysis rather than administrative data sources (e.g., DHIS2) or alternative sources (e.g., WUENIC estimates). Because the primary goal of this study was to be able to directly compare national and subnational levels and trends in no-DTP across countries, IHME estimates provided the greatest number of countries with subnational estimates for the full time period (2000–2019). 

Fifth, DTP estimates draw from household surveys and other data sources in which groups or communities with higher rates of unvaccinated children may be systematically under-represented (e.g., displaced or highly mobile populations). Accordingly, current no-DTP estimates may not fully capture the ‘true’ magnitude or trends in zero-dose children among populations with disproportionately high vulnerabilities and risks for not being vaccinated. 

Sixth, the time period of analysis focused on 2000 to 2019, and thus the identification of potential exemplars may be sensitive to estimated levels of childhood vaccination at either end of the 19-year range. Importantly, this analysis does not account for the ongoing effects of the COVID-19 pandemic, of which has had differential impacts across countries and communities since March 2020 [3,60,61]. Relatedly, these analyses do not reflect improvements in or worsening of conflict since 2019, such as in the Tigray region in Ethiopia [62].

Lastly, these analyses currently lack deeper contextual information from and by the communities most affected by higher rates of un- and under-vaccination. Our aim is to receive critical feedback on the potential applications of these positive-outlier methods for cross-country learning and synthesis around what works to reduce high rates of zero-dose prevalence, and to work with country and regional leadership to improve these approaches going forward.

## 5. Conclusions

Recognizing where exceptional progress in reducing zero-dose children has occurred is the first step toward better understanding what countries did to attain such improvements. Such insights then can inform strategy adaptions to other settings, and further reinforce successful strategies in places that achieved large reductions historically but still have large populations of unvaccinated children today. Characterizing pathways to greater vaccination equity, as well ensuring mechanisms by which effective knowledge translation and cross-country learning can be supported, will strengthen efforts toward ensuring all children can fully benefit from vaccines.

## Figures and Tables

**Table 1:** Included countries for identifying potential exemplars in reducing zero-dose children. * Gavi-supported indicates that the country received Gavi support as of 2018 or had a dedicated country hub page. ** Countries with national and subnational DTP1 estimates (for both first and second administrative units) as modeled by the Institute for Health Metrics and Evaluation. Supplementary Table S1 provides the list of initial countries considered but excluded due to not meeting inclusion criteria.

Country	World Bank FY20 Income Group	Gavi-Supported *	National and Subnational DTP1 Estimates Available, 2000–2019 **	Gavi zero-Dose Segmentation Grouping
Afghanistan	Low-income	Yes	Yes	Conflict/fragile
Angola	Lower-middle income	Yes	Yes	Core—ESA (Priority)
Bangladesh	Lower-middle income	Yes	Yes	Core—Rest of World (Priority)
Benin	Low-income	Yes	Yes	Core—WCA (Priority)
Burkina Faso	Low-income	Yes	Yes	Core—WCA (Priority)
Burundi	Low-income	Yes	Yes	Core—ESA (Standard)
Cambodia	Lower-middle income	Yes	Yes	Core—Rest of World (Standard)
Cameroon	Lower-middle income	Yes	Yes	Core—WCA (Priority)
Central African Rep	Low-income	Yes	Yes	Conflict/fragile
Chad	Low-income	Yes	Yes	Conflict/fragile
Comoros	Lower-middle income	Yes	Yes	Core—ESA (Standard)
Congo	Lower-middle income	Yes	Yes	Core—WCA (Priority)
Côte d’Ivoire	Lower-middle income	Yes	Yes	Core—WCA (Priority)
Dem Rep of the Congo	Low-income	Yes	Yes	High impact
Djibouti	Lower-middle income	Yes	Yes	Core—ESA (Priority)
Eritrea	Low-income	Yes	Yes	Core—ESA (Standard)
Ethiopia	Low-income	Yes	Yes	High impact
Gambia	Low-income	Yes	Yes	Core—WCA (Standard)
Ghana	Lower-middle income	Yes	Yes	Core—WCA (Priority)
Guinea	Low-income	Yes	Yes	Core—WCA (Priority)
Guinea-Bissau	Low-income	Yes	Yes	Core—WCA (Priority)
Haiti	Low-income	Yes	Yes	Conflict/fragile
India	Lower-middle income	Yes	Yes	High impact
Kenya	Lower-middle income	Yes	Yes	Core—ESA (Priority)
Kyrgyzstan	Lower-middle income	Yes	Yes	Core—Rest of World (Standard)
Laos	Lower-middle income	Yes	Yes	Core—Rest of World (Priority)
Lesotho	Lower-middle income	Yes	Yes	Core—ESA (Standard)
Liberia	Low-income	Yes	Yes	Core—WCA (Standard)
Madagascar	Low-income	Yes	Yes	Core—ESA (Priority)
Malawi	Low-income	Yes	Yes	Core—ESA (Priority)
Mali	Low-income	Yes	Yes	Conflict/fragile
Mauritania	Lower-middle income	Yes	Yes	Core—WCA (Standard)
Mozambique	Low-income	Yes	Yes	Core—ESA (Priority)
Myanmar	Lower-middle income	Yes	Yes	Core—Rest of World (Priority)
Nepal	Low-income	Yes	Yes	Core—Rest of World (Priority)
Niger	Low-income	Yes	Yes	Conflict/fragile
Nigeria	Lower-middle income	Yes	Yes	High impact
Pakistan	Lower-middle income	Yes	Yes	High impact
Papua New Guinea	Lower-middle income	Yes	Yes	Conflict/fragile
Rwanda	Low-income	Yes	Yes	Core—ESA (Standard)
São Tomé and Príncipe	Lower-middle income	Yes	Yes	Core—WCA (Standard)
Senegal	Lower-middle income	Yes	Yes	Core—WCA (Standard)
Sierra Leone	Low-income	Yes	Yes	Core—WCA (Standard)
Somalia	Low-income	Yes	Yes	Conflict/fragile
South Sudan	Low-income	Yes	Yes	Conflict/fragile
Sudan	Lower-middle income	Yes	Yes	Conflict/fragile
Tajikistan	Low-income	Yes	Yes	Core—Rest of World (Standard)
Tanzania	Low-income	Yes	Yes	Core—ESA (Priority)
Timor-Leste	Lower-middle income	Yes	Yes	Core—Rest of World (Standard)
Togo	Low-income	Yes	Yes	Core—WCA (Priority)
Uganda	Low-income	Yes	Yes	Core—ESA (Priority)
Uzbekistan	Lower-middle income	Yes	Yes	Core—Rest of World (Standard)
Vietnam	Lower-middle income	Yes	Yes	Core—Rest of World (Standard)
Yemen	Low-income	Yes	Yes	Conflict/fragile
Zambia	Lower-middle income	Yes	Yes	Core—ESA (Priority)
Zimbabwe	Lower-middle income	Yes	Yes	Core—ESA (Standard)

**Table 2:** Comparing levels and changes in no-DTP prevalence, nationally and for subnational gaps, from 2000 to 2019 for 56 LMICs. Bolded countries are those with the largest progress in reducing zero-dose children for both national and subnational gaps, based on the difference between the 5th and 95th percentiles for no-DTP prevalence at the second-level administrative unit, for either absolute or relative declines from 2000 to 2019. pp = percentage-points.

Country	National Prevalence of No-DTP				Subnational Gap in No-DTP Prevalence (5–95th Percentile Difference across Districts)			
	2000 (%)	2019 (%)	Absolute Change, 2000–2019 (pp)	Relative Change, 2000–2019 (%)	2000 (pp)	2019 (pp)	Absolute Change, 2000–2019 (pp)	Relative Change, 2000–2019 (%)
Afghanistan	53.4	14.5	−39.0	−72.9	39.0	31.8	−7.2	−18.5
Angola	46.3	24.8	−21.5	−46.4	38.6	37.2	−1.3	−3.5
**Bangladesh**	**8.8**	**0.2**	**−8.6**	**−98.0**	**14.4**	**0.0**	**−14.4**	**−99.9**
Benin	11.3	13.6	2.4	21.1	16.9	19.9	3.0	18.0
Burkina Faso	23.8	4.6	−19.2	−80.6	31.2	10.7	−20.5	−65.8
**Burundi**	**18.4**	**2.3**	**−16.0**	**−87.3**	**17.1**	**2.4**	**−14.7**	**−85.9**
Cambodia	22.8	7.3	−15.6	−68.2	27.2	18.6	−8.6	−31.6
Cameroon	26.4	17.4	−9.0	−34.1	48.7	25.8	−22.9	−47.0
Central African Rep	44.0	28.9	−15.1	−34.3	19.1	19.7	0.6	3.1
Chad	59.7	24.0	−35.6	−59.7	40.4	36.4	−4.0	−10.0
Comoros	19.1	6.8	−12.3	−64.6	7.5	3.2	−4.3	−57.5
Congo	48.5	33.2	−15.3	−31.5	22.2	30.6	8.4	38.1
Cote d’Ivoire	17.9	2.3	−15.6	−87.1	20.5	4.4	−16.1	−78.4
**Dem Rep of the Congo**	**51.9**	**6.9**	**−45.0**	**−86.8**	**46.8**	**16.0**	**−30.8**	**−65.8**
Djibouti	34.3	26.1	−8.2	−24.0	17.2	18.0	0.7	4.4
Eritrea	10.6	1.1	−9.4	−89.2	15.3	3.8	−11.4	−74.8
**Ethiopia**	**63.4**	**11.7**	**−51.6**	**−81.5**	**47.9**	**30.8**	**−17.2**	**−35.8**
Gambia	8.6	3.8	−4.8	−55.7	9.4	4.2	−5.2	−55.7
Ghana	12.5	6.5	−6.0	−48.2	26.2	5.6	−20.6	−78.7
Guinea	35.6	38.4	2.8	7.9	33.2	39.3	6.1	18.4
Guinea-Bissau	19.5	6.6	−12.9	−66.3	13.4	7.8	−5.6	−41.7
Haiti	20.5	21.0	0.6	2.8	17.9	9.7	−8.2	−45.7
**India**	**30.9**	**7.2**	**−23.6**	**−76.5**	**52.5**	**19.7**	**−32.7**	**−62.4**
Kenya	9.6	10.0	0.4	4.4	16.6	20.8	4.1	24.8
Kyrgyzstan	10.3	5.0	−5.3	−51.5	7.0	5.0	−2.1	−29.3
Laos	30.8	24.6	−6.1	−19.9	28.4	21.5	−6.9	−24.4
Lesotho	13.1	9.6	−3.5	−26.9	4.3	3.3	−1.1	−24.6
Liberia	21.2	11.2	−10.0	−47.2	37.9	12.3	−25.6	−67.6
Madagascar	25.7	10.7	−15.0	−58.5	34.0	17.1	−16.9	−49.8
Malawi	4.8	3.4	−1.4	−29.2	12.0	2.6	−9.4	−78.4
Mali	36.4	17.1	−19.3	−53.1	47.8	40.5	−7.3	−15.2
Mauritania	28.1	6.5	−21.6	−76.8	33.0	10.1	−22.8	−69.3
Mozambique	12.7	3.6	−9.1	−71.8	23.5	7.6	−15.9	−67.5
Myanmar	11.5	17.6	6.1	52.9	25.4	13.7	−11.7	−46.0
Nepal	13.5	8.7	−4.7	−35.2	24.4	7.7	−16.7	−68.4
Niger	52.6	18.2	−34.5	−65.4	23.5	20.4	−3.2	−13.4
Nigeria	55.5	28.9	−26.6	−48.0	71.7	64.9	−6.8	−9.5
Pakistan	24.0	8.4	−15.6	−65.1	37.2	36.5	−0.7	−2.0
Papua New Guinea	17.3	44.4	27.1	156.4	13.7	32.5	18.8	137.7
Rwanda	5.7	1.9	−3.8	−66.9	6.9	1.3	−5.6	−81.5
São Tomé and Príncipe	14.6	3.0	−11.6	−79.5	3.6	4.0	0.4	11.4
Senegal	17.3	2.2	−15.1	−87.1	23.9	9.9	−14.0	−58.4
Sierra Leone	31.1	9.0	−22.1	−71.0	30.5	7.5	−23.0	−75.4
Somalia	51.2	51.1	−0.1	−0.2	44.0	27.9	−16.1	−36.5
South Sudan	64.1	33.0	−31.1	−48.5	25.2	22.5	−2.7	−10.6
Sudan	33.1	3.4	−29.7	−89.6	14.4	4.8	−9.6	−66.9
Tajikistan	8.9	6.4	−2.5	−28.0	6.3	8.4	2.1	32.7
Tanzania	8.3	5.7	−2.7	−32.0	20.7	8.5	−12.2	−59.0
Timor-Leste	51.3	27.0	−24.3	−47.4	18.6	15.3	−3.3	−17.9
Togo	23.1	13.1	−10.1	−43.5	26.1	11.1	−15.0	−57.5
Uganda	21.9	6.7	−15.2	−69.2	29.2	6.3	−22.9	−78.5
Uzbekistan	1.3	7.2	5.9	441.9	2.9	5.1	2.2	77.5
Vietnam	7.8	4.6	−3.2	−41.0	17.3	8.7	−8.7	−50.0
Yemen	27.1	22.4	−4.7	−17.2	43.4	35.4	−8.1	−18.5
Zambia	7.5	5.8	−1.7	−22.3	10.2	7.1	−3.1	−30.5
Zimbabwe	20.1	5.5	−14.7	−72.9	19.5	6.6	−12.9	−66.0

## Data Availability

Vaccine estimates used in this analysis are currently unpublished estimates from the Institute for Health Metrics and Evaluation (IHME). Data may be shared upon request to the corresponding author and IHME.

## Supplementary Materials

The following supporting information can be downloaded at: https://www.mdpi.com/article/10.3390/vaccines11030647/s1, Table S1. Initial countries considered for the present analysis but were excluded for not meeting inclusion criteria; Figure S1. National and subnational trends in the prevalence of no-DTP children, 2000-2019, in the Democratic Republic of the Congo (A), Ethiopia (B), India (C), Bangladesh (D), and Burundi (E); Figure S2. Subnational no-DTP prevalence in Nigeria (A) and Ethiopia (B), 2000–2019.
