# Supplementary material for: Assessing Potential Exemplars in Reducing Zero-Dose Children: A Novel Approach for Identifying Positive Outliers in Decreasing National Levels and Geographic Inequalities in Unvaccinated Children
Source: Vaccines (Basel). 2023 Mar 14;11(3):647. doi: 10.3390/vaccines11030647 (PMC10056845; doi:10.3390/vaccines11030647)
Supplement: Supplementary file 1 [file vaccines-11-00647-s001.zip › vaccines-2226348-supplementary.pdf]

Supplementary Material

Title: Assessing potential exemplars in reducing zero-dose children: a novel approach for identifying positive outliers in decreasing national levels and geographic inequalities in unvaccinated children

Supplementary Table S1. Initial countries considered for the present analysis but were excluded for not meeting inclusion criteria. *Gavi-supported indicates that the country received Gavi support as of 2018 or had a dedicated country hub page. **Countries with national and subnational DTP1 estimates (for both first and second administrative units) as modelled by the Institute for Health Metrics and Evaluation. Table 1 lists the 56 countries included in the present analysis.

Reason for exclusion	Country	World Bank FY20 income group	Gavi-supported*	National and subnational DTP1 estimates available, 2000-2019**	Gavi zero-dose segmentation grouping
Excluded due to post-transition MIC status or omission from Gavi zero-dose segmentation groups (*n*=13)	Armenia	Upper-middle income	Yes	Yes	Post-transition MIC
	Azerbaijan	Upper-middle income	Yes	Yes	Post-transition MIC
	Bolivia	Lower-middle income	Yes	Yes	Post-transition MIC
	Cuba	Upper-middle income	Yes	Yes	Post-transition MIC
	Guyana	Upper-middle income	Yes	Yes	Post-transition MIC
	Honduras	Lower-middle income	Yes	Yes	Post-transition MIC
	Indonesia	Lower-middle income	Yes	Yes	Post-transition MIC
	Moldova	Lower-middle income	Yes	Yes	Post-transition MIC
	Mongolia	Lower-middle income	Yes	Yes	Post-transition MIC
	Nicaragua	Lower-middle income	Yes	Yes	Post-transition MIC
	Sri Lanka	Upper-middle income	Yes	Yes	Post-transition MIC
	Turkmenistan	Upper-middle income	Yes	Yes	Omitted
	Ukraine	Lower-middle income	Yes	Yes	Omitted
Excluded due to lack of subnational DTP1 estimates for 2000-2019 (*n*=19)	Albania	Upper-middle income	Yes	No	
	Bhutan	Lower-middle income	Yes	No	
	Bosnia and Herzegovina	Upper-middle income	Yes	No	
	Cabo Verde	Lower-middle income	No	No	
	China	Upper-middle income	Yes	No	
	Egypt	Lower-middle income	No	No	
	El Salvador	Lower-middle income	No	No	
	Eswatini	Lower-middle income	No	No	
	Georgia	Upper-middle income	Yes	No	
	Kiribati	Lower-middle income	Yes	No	
	Micronesia	Lower-middle income	No	No	
	Morocco	Lower-middle income	No	No	
	North Korea	Low-income	Yes	No	
	Palestine	Lower-middle income	No	No	
	Philippines	Lower-middle income	No	No	

Reason for exclusion	Country	World Bank FY20 income group	Gavi-supported*	National and subnational DTP1 estimates available, 2000-2019**	Gavi zero-dose segmentation grouping
	Solomon Islands	Lower-middle income	Yes	No	
	Syria	Low-income	No	No	
	Tunisia	Lower-middle income	No	No	
	Vanuatu	Lower-middle income	No	No	
Excluded due to being classified as upper-middle income by World Bank for FY20 *and* not Gavi-supported* (n=47)	Algeria	Upper-middle income	No	No	
	American Samoa	Upper-middle income	No	No	
	Argentina	Upper-middle income	No	No	
	Belarus	Upper-middle income	No	No	
	Belize	Upper-middle income	No	No	
	Botswana	Upper-middle income	No	No	
	Brazil	Upper-middle income	No	No	
	Bulgaria	Upper-middle income	No	No	
	Colombia	Upper-middle income	No	No	
	Costa Rica	Upper-middle income	No	No	
	Dominica	Upper-middle income	No	No	
	Dominican Republic	Upper-middle income	No	No	
	Ecuador	Upper-middle income	No	No	
	Equatorial Guinea	Upper-middle income	No	No	
	Fiji	Upper-middle income	No	No	
	Gabon	Upper-middle income	No	No	
	Grenada	Upper-middle income	No	No	
	Guatemala	Upper-middle income	No	No	
	Iran	Upper-middle income	No	No	
	Iraq	Upper-middle income	No	No	
	Jamaica	Upper-middle income	No	No	
	Jordan	Upper-middle income	No	No	
	Kazakhstan	Upper-middle income	No	No	
	Lebanon	Upper-middle income	No	No	
	Libya	Upper-middle income	No	No	
	Malaysia	Upper-middle income	No	No	
	Maldives	Upper-middle income	No	No	
	Marshall Islands	Upper-middle income	No	No	
	Mauritius	Upper-middle income	No	No	
	Mexico	Upper-middle income	No	No	
	Montenegro	Upper-middle income	No	No	
	Namibia	Upper-middle income	No	No	
	North Macedonia	Upper-middle income	No	No	
	Paraguay	Upper-middle income	No	No	
	Peru	Upper-middle income	No	No	
	Romania	Upper-middle income	No	No	

Reason for exclusion	Country	World Bank FY20 income group	Gavi-supported*	National and subnational DTP1 estimates available, 2000-2019**	Gavi zero-dose segmentation grouping
	Russia	Upper-middle income	No	No	
	Saint Lucia	Upper-middle income	No	No	
	Saint Vincent and the Grenadines	Upper-middle income	No	No	
	Samoa	Upper-middle income	No	No	
	Serbia	Upper-middle income	No	No	
	South Africa	Upper-middle income	No	No	
	Suriname	Upper-middle income	No	No	
	Thailand	Upper-middle income	No	No	
	Tonga	Upper-middle income	No	No	
	Turkey	Upper-middle income	No	No	
	Venezuela	Upper-middle income	No	No	

Supplementary Figure S1. National and subnational trends in the prevalence of no-DTP children, 2000-2019, in the Democratic Republic of the Congo (A), Ethiopia (B) India (C) Bangladesh (D) and Burundi (E) Darker shaded and larger circles represent the national estimate of no-DTP prevalence for a given country, while the lighter shaded and smaller circles represent estimates of no-DTP prevalence for second-level administrative unit for a given country. All estimates were provided by the Institute for Health Metrics and Evaluation.

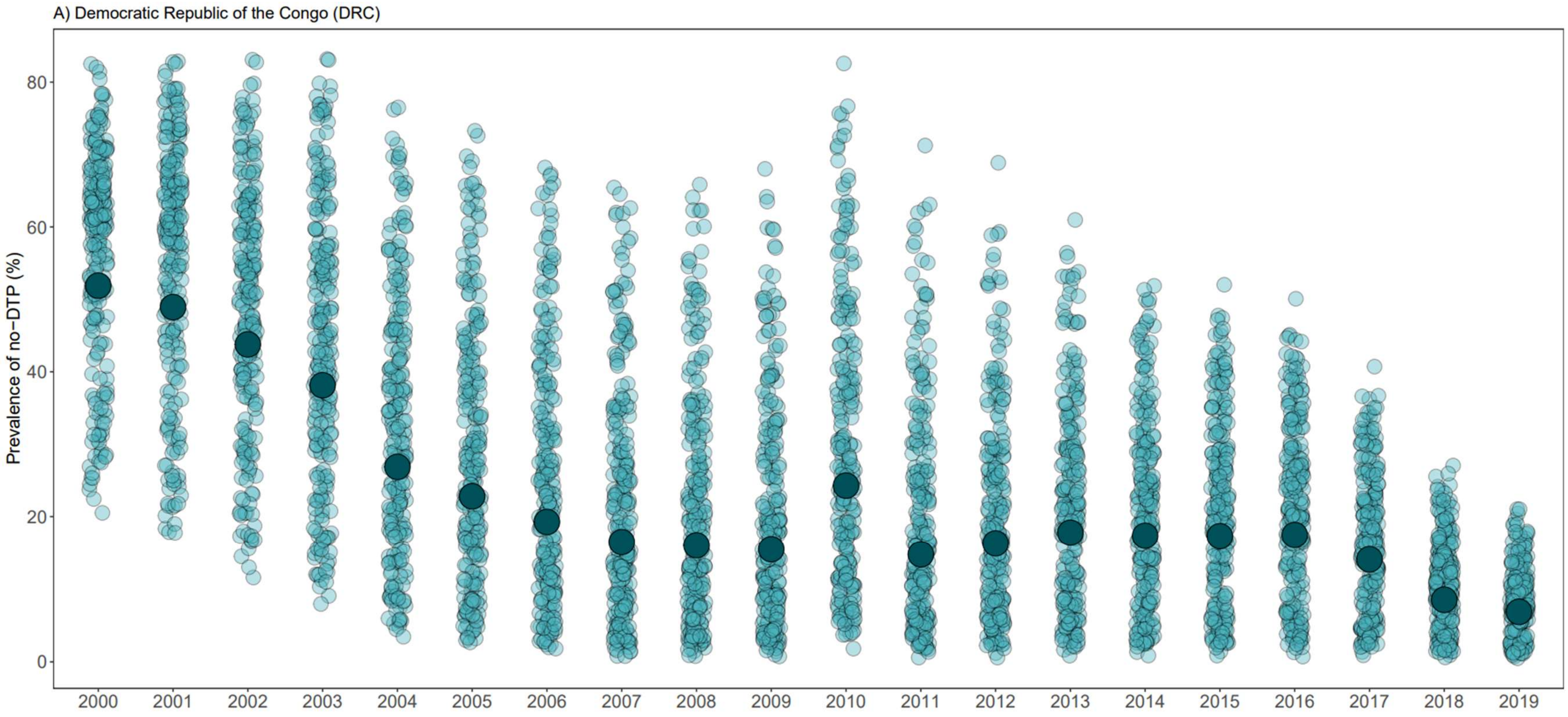

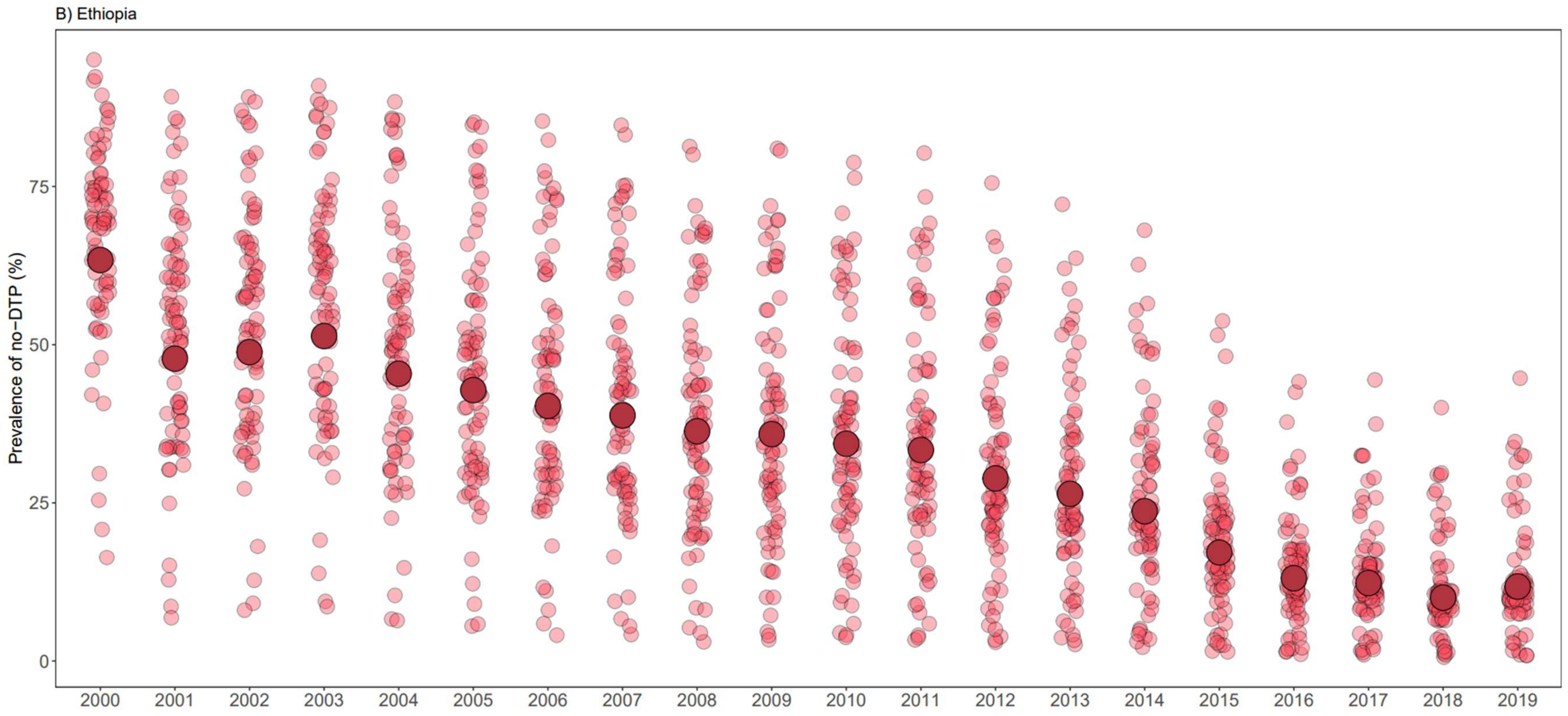

B) Ethiopia
Prevalence of no-DTP (%)
0
25
50
75
2000
2001
2002
2003
2004
2005
2006
2007
2008
2009
2010
2011
2012
2013
2014
2015
2016
2017
2018
2019

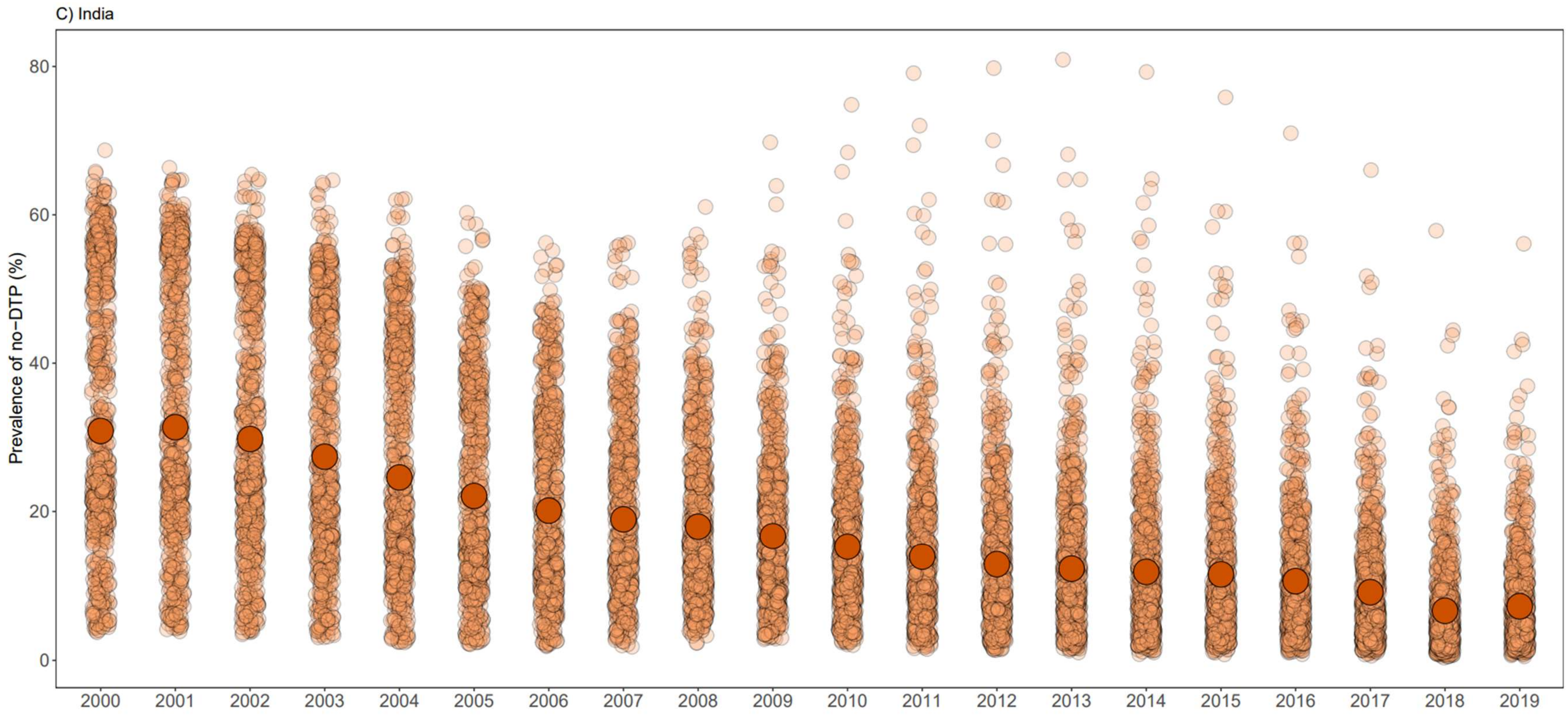

C) India
Prevalence of no-DTP (%)
0
20
40
60
80
2000
2001
2002
2003
2004
2005
2006
2007
2008
2009
2010
2011
2012
2013
2014
2015
2016
2017
2018
2019

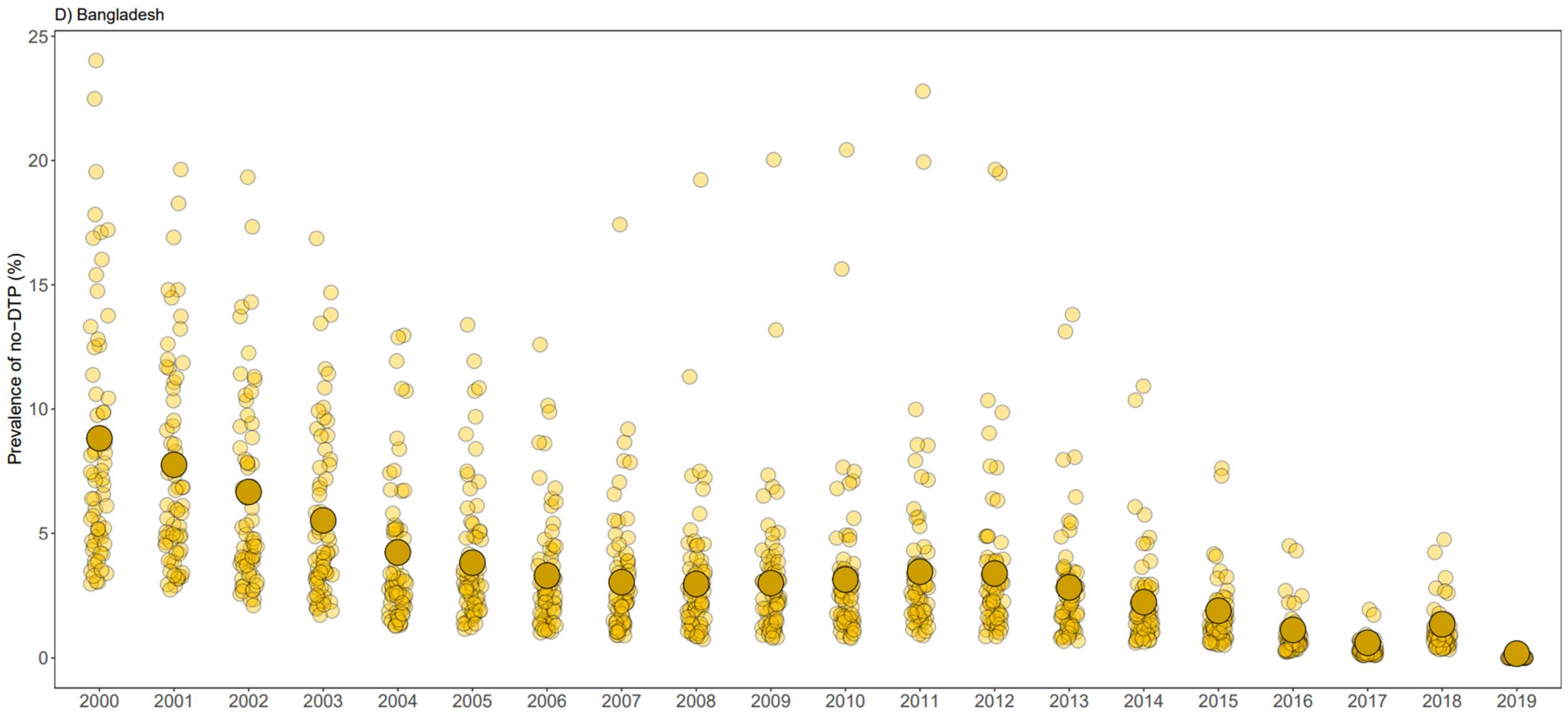

D) Bangladesh
Prevalence of no-DTP (%)
25
20
15
10
5
0
2000
2001
2002
2003
2004
2005
2006
2007
2008
2009
2010
2011
2012
2013
2014
2015
2016
2017
2018
2019

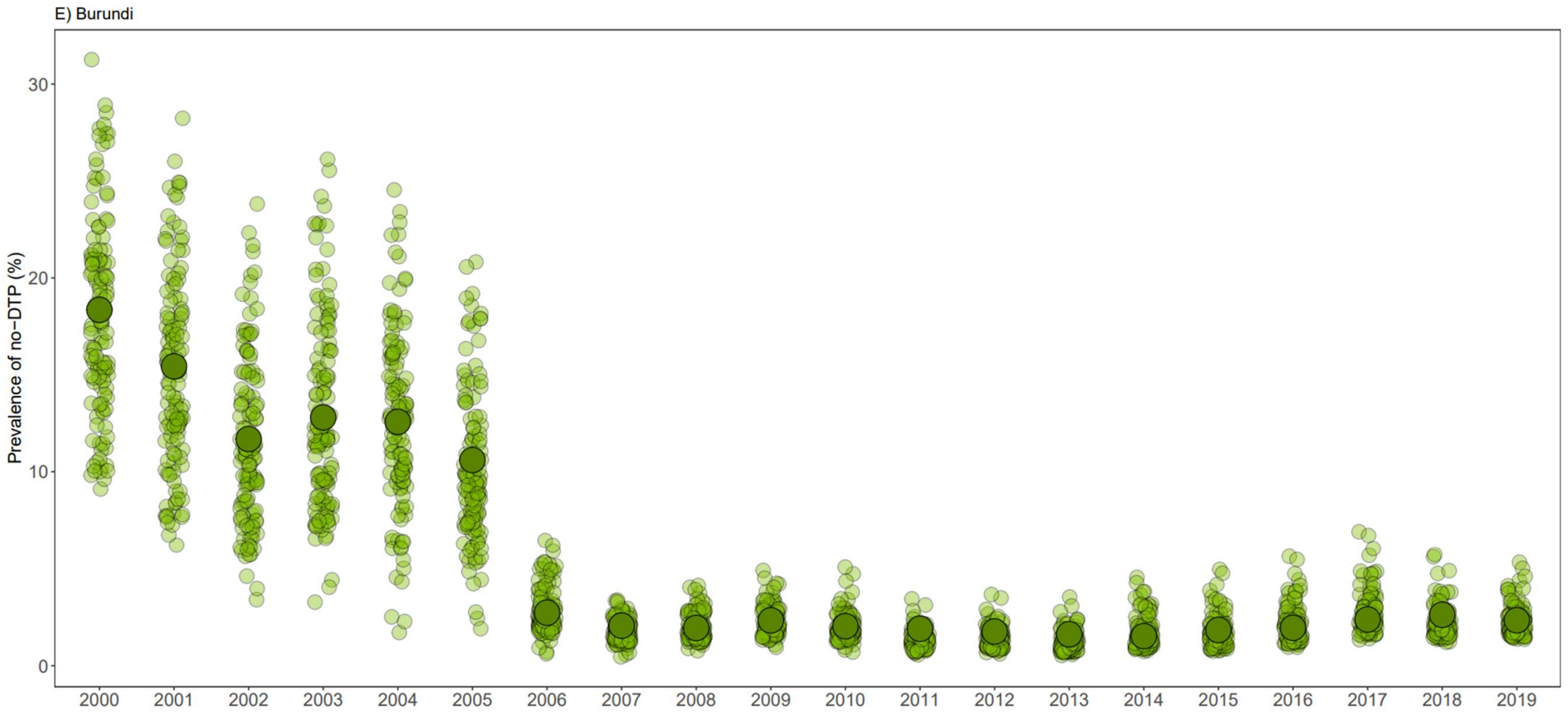

E) Burundi
Prevalence of no−DTP (%)
0
10
20
30
2000
2001
2002
2003
2004
2005
2006
2007
2008
2009
2010
2011
2012
2013
2014
2015
2016
2017
2018
2019

Supplementary Figure S2. Subnational no-DTP prevalence in Nigeria (A)and Ethiopia (B),2000-2019. Select trends for first-level administrative units are color-coded in shades of maroon, teal, green, and yellow while zone-level trends are light gray in color. All estimates were provided by the Institute for Health Metrics and Evaluation.

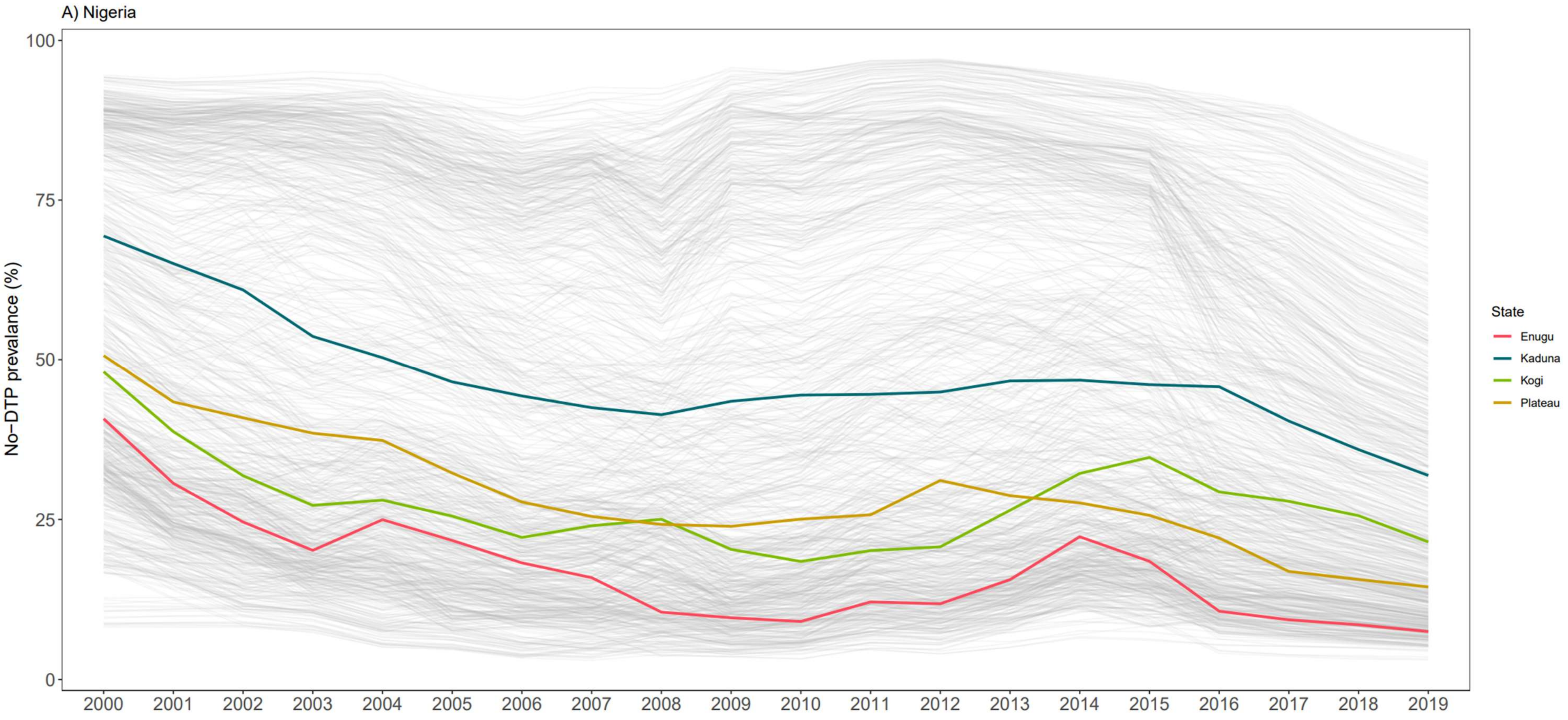

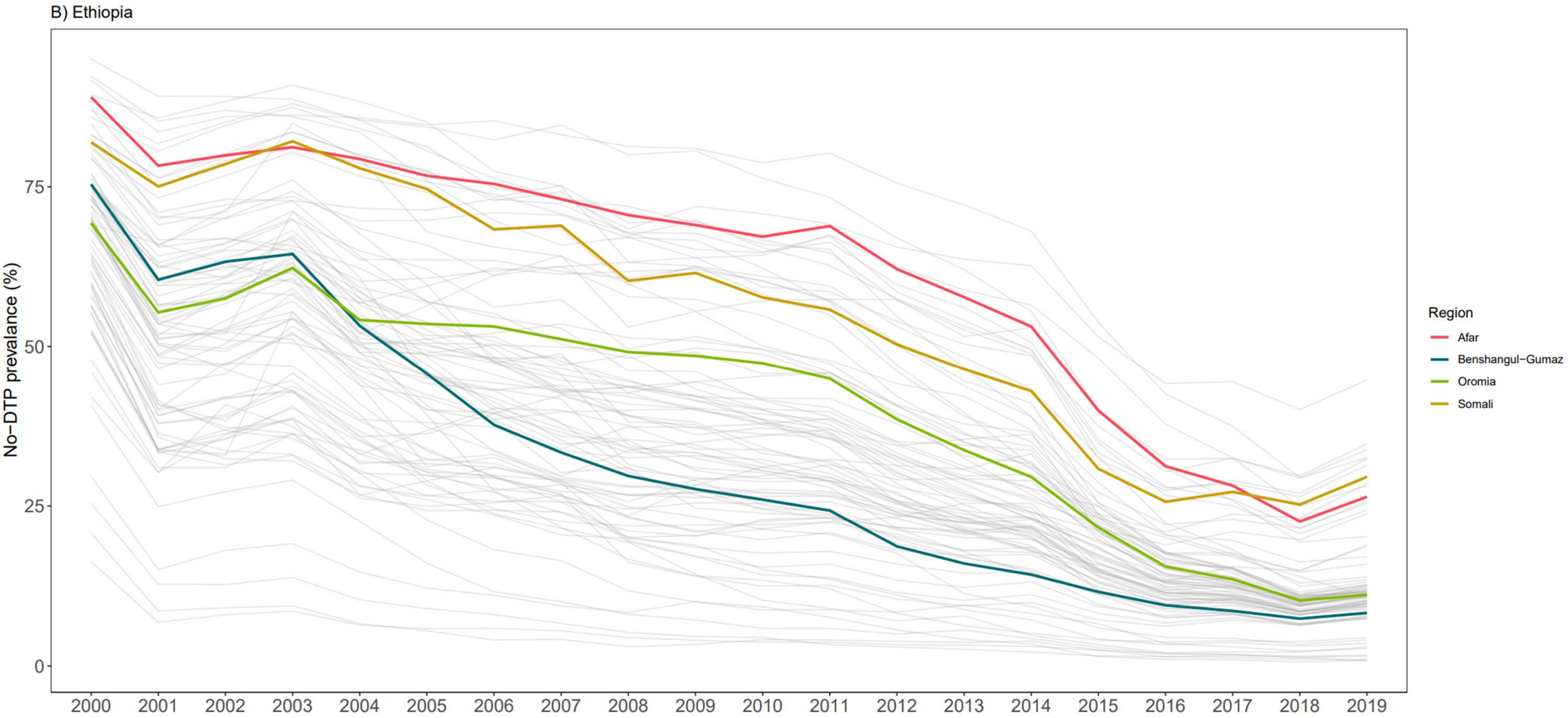
B) Ethiopia
No−DTP prevalance (%)
0
25
50
75
2000
2001
2002
2003
2004
2005
2006
2007
2008
2009
2010
2011
2012
2013
2014
2015
2016
2017
2018
2019
Region
Afar
Benshangul−Gumaz
Oromia
Somali
